# Topical Photodynamic Therapy in a Medical Centre: The Scottish Dermatology Experience

**DOI:** 10.1111/phpp.70010

**Published:** 2025-02-03

**Authors:** Chayada Chaiyabutr, Robert Dawe, Andrea Lesar, Sally H. Ibbotson

**Affiliations:** ^1^ Department of Dermatology, Faculty of Medicine Siriraj Hospital Mahidol University Bangkok Thailand; ^2^ Scottish Photodynamic Therapy Centre, Photobiology Unit University of Dundee & NHS Tayside, Ninewells Hospital & Medical School Dundee UK

**Keywords:** actinic keratoses, basal cell carcinoma, Bowen's disease, daylight, photodynamic therapy

## Abstract

**Background:**

Topical photodynamic therapy (PDT) is widely used in dermatology for treating superficial non‐melanoma skin cancer (NMSC) and dysplasia. This study aims to assess real‐world outcomes of PDT in a Scottish dermatology service.

**Methods:**

We retrospectively reviewed patients with superficial NMSC and dysplasia who underwent conventional and daylight PDT at the Photobiology Unit, Dundee, Scotland.

**Results:**

A total of 705 patients with 2108 NMSC and precancerous skin lesions underwent conventional PDT. Clearance at 12 months was achieved in 53.4% of actinic keratoses (AK), 71.3% of Bowenoid AK, 86.4% of Bowen's disease (BD), 89.0% of superficial basal cell carcinoma (BCC), and 89.7% of nodular BCC. On multivariate analysis, small lesion size and thin histological tumour thickness of superficial BCC were features, which were associated with likelihood of achieving clearance after PDT. Female sex, head/neck sites, larger lesion size, strong pre‐treatment fluorescence intensity, fluorescence specificity, prominent treatment‐induced erythema and an urticarial reaction were associated with moderate to severe pain during PDT. Daylight PDT for 77 AK patients (158 treatments) showed excellent or good outcomes in 63.3% of lesions. Higher visible light exposure is correlated with better treatment outcomes.

**Conclusions:**

In real‐life settings, whilst the PDT response rates of BD and selected BCC are high and consistent with clinical trial outcomes, the efficacy rates for AK appear lower than expected. This emphasizes the need for realistic expectations in chronic disease management. Through review over a prolonged period, factors associated with PDT tolerability and outcomes were identified, allowing predictive utilisation for optimizing patient‐centred PDT regimens.

## 1 Introduction

Topical photodynamic therapy (PDT) has been increasingly used in dermatology since 1990 and employs the application of topical photosensitiser pro‐drugs prior to visible light exposure [1, 2]. The main indications for use are superficial non‐melanoma skin cancers (NMSC), namely superficial basal cell carcinoma (BCC), Bowen's disease (BD) and actinic keratosis (AK) [3]. Currently, there are three photosensitiser pro‐drugs licensed for use in topical PDT: methyl aminolaevulinate (MAL; Metvix), a nanoemulsion of 5‐aminolaevulinic acid (ne‐ALA; Ameluz) and in the US, ALA as Levulan Kerastick [3].

Topical PDT had been widely used for AK, with > 70% complete response rates and excellent cosmetic outcomes [4, 5, 6]. It is also an excellent treatment for BD, particularly on the lower legs, and for superficial BCC, with high efficacy rates (> 80%) observed in published studies [7, 8]. It may also be effective for thin‐nodular BCC if other conventional approaches are considered suboptimal [3, 4]. However, hospital‐based conventional PDT (cPDT) can be difficult to undertake large area treatments and multiple lesions as it may be inconvenient, requiring multiple treatment sessions and can be painful if large fields are treated. In the case of field change AK, daylight PDT (dPDT) has been increasingly used as an effective and well‐tolerated treatment and is licensed for superficial face and scalp AK [4]. The primary objective of this study was to retrospectively review the efficacy and tolerability of topical cPDT and dPDT in Scotland when used in routine “real‐time” clinical practice. The secondary objectives over this long‐term review were to determine whether there are factors associated with PDT outcomes and PDT‐induced pain.

## 2 Material and Methods

This study was a retrospective review of patients with AK, BD and BCC who were treated with cPDT between 2009 and April 2022 and AK with dPDT between 2016 and 2021 in a teaching hospital setting at Photobiology Unit, Ninewells Hospital & Medical School, Scotland. Notably, the early data relating to a small subset of these patients was presented previously, but the current study includes a much larger cohort, a wider range of diagnoses and longer follow‐up [5, 9]. Demographic information, diagnosis, lesion characteristics, treatment protocols, outcomes and adverse effects were retrieved through patient medical records and an in‐house database. NHS Tayside Caldicott Guardian approval was obtained for this study.

Most of the lesions treated with cPDT (91.5%) had received a range of previous therapies and were considered to be treatment‐resistant, with relapse or failure to respond to previous treatments. The patients receiving cPDT for AK had been referred for field‐change treatment. Surface preparation of lesions was initially undertaken using a disposable ring curette (Stiefel, UK) to remove hyperkeratosis, followed by application of the photosensitiser pro‐drug (MAL; Metvix, ne‐ALA; Ameluz, or ALA [5‐ALA, 20% w/v in UngM]), typically for 3 h with occlusion (Tegaderm, UK). This was followed by irradiation using a red‐light light‐emitting diode (LED). The dose of red light use is depended on the diagnosis (AK 37.5 or 75 J/cm^2^ and typically 75 J/cm^2^ for other diagnoses). Pre‐treatment fluorescence intensity and specificity were assessed visually using Wood's light, prior to irradiation (Figure S1). Fluorescence intensity was graded as 0–3 for none, mild, moderate and strong fluorescence, while fluorescence specificity was assessed as specific to the target lesion or not (yes/no). Phototoxicity, assessed visually as erythema, was graded immediately after cPDT as 0–3 for none, mild, moderate or severe. Maximal pain experienced during treatment was determined immediately after cPDT using a patient‐recorded visual analogue scale or numerical rating score (0–10). If there was intolerable pain during cPDT, a fan, cold air cooling or local anesthetic drugs were occasionally used. For lesions receiving multiple treatments, treatment‐related reactions (erythema, urticaria) and pain scores for the first treatment were analysed.

For patients with AK and Bowenoid AK (lesions which clinically and/or histologically had overlapping features of AK and BD), one cPDT session per treatment cycle was undertaken, while for BCC and BD two cPDT sessions were undertaken 1 week apart from the first treatment cycle. Outcomes were determined by clinical assessment at 3 months after the first cPDT treatment cycle, with repeat cPDT undertaken if only a partial response had been achieved. Follow‐up was up to 12 months after the last cPDT treatment cycle.

Most patients who were referred for field‐directed dPDT for AK had either failed to respond or had relapsed after other treatments—usually 5‐fluorouracil (Efudix or Actikerall), imiquimod (Aldara) and/or diclofenac‐hyaluronan (Solaraze). dPDT was undertaken between April and October. Surface preparation of lesions was initially undertaken using a disposable ring curette (Stiefel, UK) to remove any prominent hyperkeratosis, followed by application of absorbent sunscreen (Actinica or LaRoche Posay Anthelios), which was applied to all exposed areas. Once dry a few minutes later, the photosensitiser pro‐drug (MAL or ne‐ALA) was applied thinly to all AK treatment fields—mainly on face and scalp and left unoccluded. Patients were advised to expose the treatment areas to unshaded daylight, within 45 min of pro‐drug application, and to have continuous daylight exposure for 2.5 h in their own environment, such as a garden or when out walking. If it was a rainy day, patients could be treated using light through window glass by sitting next to a large window at home and extending the light exposure time to 3 h. Daylight illuminance was recorded using portable light meters, photodiodes (HOBO), which were pinned to their clothing at an exposed site.

Patients generally received 1–4 dPDT treatments per year at 6–8 weekly intervals, constituting a one‐year treatment course. Outcome was assessed clinically at the end of each year. The outcome for dPDT was categorized as excellent or good (approximately > 75% improvement), moderate (approximately ≥ 50%–75% improvement) and slight or no response (~< 50% improvement). Pain score after each treatment was determined by patient‐recorded visual analogue scale (0–10), and a mean value for each patient was determined for their treatment course.

Statistical analysis was performed using PASW Statistics for Windows (version 18; SPSS Inc., Chicago, IL, USA). Forward stepwise logistic regression was used to analyse the outcomes for clearance at 12 months and pain score ≥ 4 for cPDT. Factors with significance at *p* < 0.1 in univariate analyses were included in the multivariate analyses. A *p* value of less than 0.05 was considered statistically significant.

## 3 Results

**FIGURE 1:**
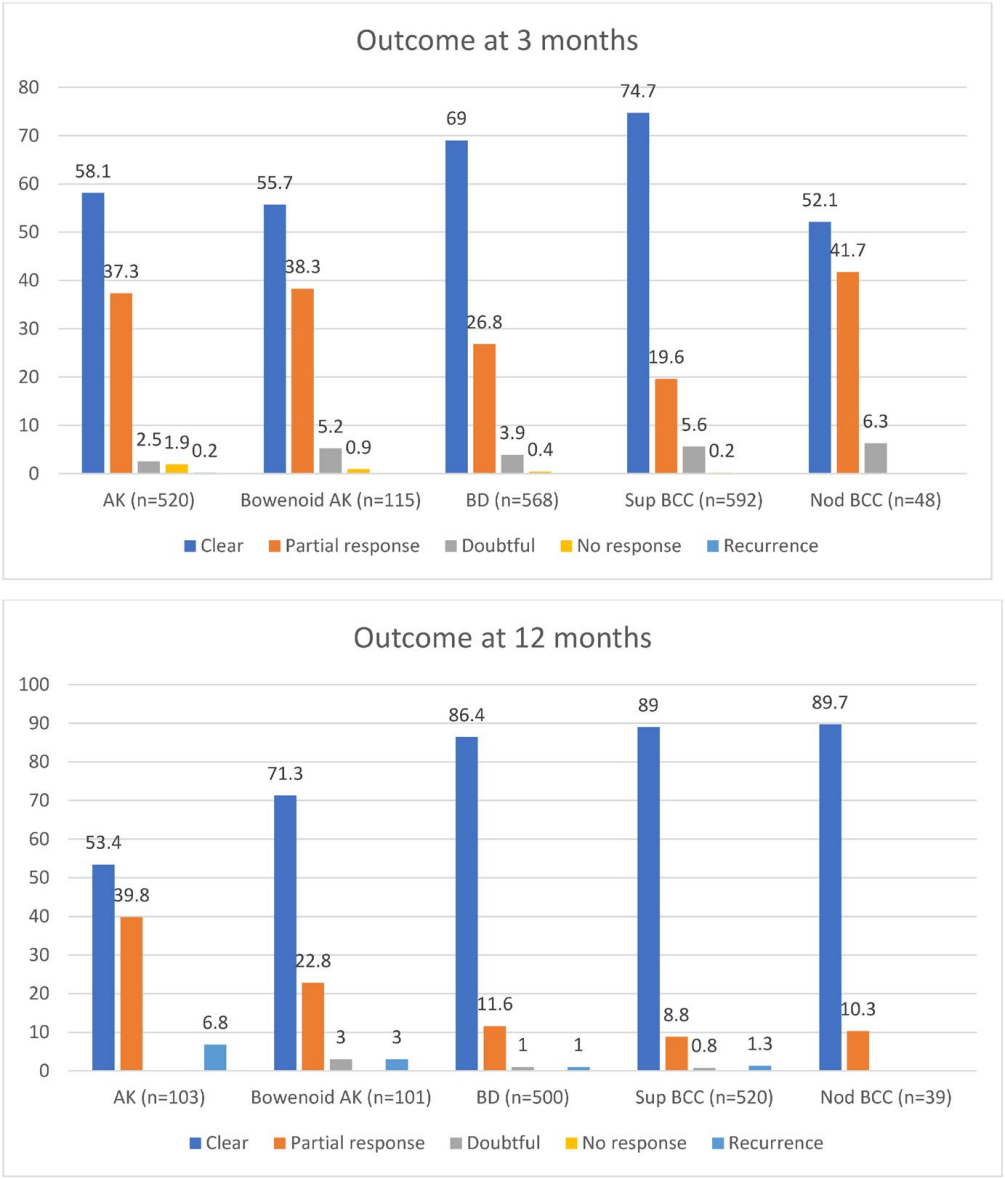
Percentage of outcome in conventional PDT at 3 and 12 months. AK, actinic keratoses; BCC, basal cell carcinoma; BD, Bowen's disease; Nod, nodular; Sup, superficial.

**Conventional PDT**: We derived data from 705 patients with 2108 lesions (AK, Bowenoid AK, BD, BCC) who had been treated with cPDT. The majority of patients were female (56.7%) and of skin phototype I‐II (81.5%). The diagnoses included 179 patients with 572 AK lesions/treatment fields, 69 patients with 157 Bowenoid AK lesions, 299 patients with 652 BD lesions, 281 patients with 670 superficial BCC lesions and 52 patients with 57 nodular BCC lesions. Some patients had multiple diagnoses. Demographic data, lesion characteristics, treatment parameters and adverse effects are shown in Table 1. Outcomes of treatment at 3 and 12 months after the first cPDT cycle, according to diagnosis, are shown in Figure 1.

**TABLE 1:** Demographic data, lesion characteristics, treatment protocol and reactions in conventional PDT.

	AK, *N* = 572		Bowenoid AK *N* = 157		BD, *N* = 652		Sup BCC, *N* = 670		Nod BCC, *N* = 57	
	*n*	%	*n*	%	*n*	%	*n*	%	*n*	%
Demographic data										
Sex										
Female	215	37.6	101	64.3	547	83.9	219	32.7	23	40.4
Male	357	62.4	56	35.7	105	16.1	451	67.3	34	59.6
Mean age (years), SD	69.2	9.6	70.6	9.5	72.5	9.0	63.6	11.6	67.5	10.0
Skin phototype I–II (*n* = 1904)	466	90.0	122	87.1	475	84.7	498	78.5	39	76.5
Immunosuppressed (*n* = 1874)	24	4.9	21	15.7	25	4.3	25	4.1	4	7.5
Lesion characteristics										
Median lesion/field size (cm), IQR (*n* = 2048)	6	3, 10	3.5	1.5, 8	1.5	1, 3	1.5	1, 2	2	1, 3
Median tumour thickness (mm), IQR (*n* = 215)							0.3	0.2, 0.5	0.9	0.7, 1.3
Location (*n* = 2089)										
Head/neck	330	57.9	59	38.3	29	4.5	90	13.6	13	23.6
Trunk	43	7.5	8	5.2	24	3.7	376	56.8	23	41.8
Extremities	197	34.6	87	56.5	595	91.8	196	29.6	19	34.5
Treatment protocol										
Photosensitizer										
ALA	67	11.7	21	13.4	38	5.8	20	3.0	2	3.5
Ne‐ALA	375	65.6	21	13.4	1	0.2	22	3.3	6	10.5
MAL	130	22.7	115	73.2	613	94	628	93.7	49	86
Fluorescence intensity (*n* = 1818)	2	1, 3	2	1, 3	2	1, 3	3	2, 3	3	2, 3
None	17	3.2	16	11.2	26	4.8	13	2.4	2	3.8
Mild	128	23.8	33	23.1	129	24.0	62	11.3	4	7.7
Moderate	186	34.6	34	23.8	180	33.5	134	24.5	18	34.6
Max	206	38.4	60	42.0	203	37.7	339	61.9	28	53.8
Fluorescence specificity (*n* = 1813)	501	93.6	127	88.8	510	95	523	95.6	49	96.1
Median red‐light dose, IQR (J/cm^2^) (*n* = 2104)	37.5	37.5, 75	75	75, 75	75	75, 75	75	75, 75	75	75, 75
Mean number of treatments, SD (*n* = 2053)	1.7	1.1	2.6	1.1	2.7	1.2	2.7	1.1	3.2	1.2
Pain relieving method										
Fan/cold air cooling (*n* = 2104)	82	14.4	31	19.7	93	14.3	82	12.3	6	10.5
Local anesthetic drug	24	4.2	16	10.2	32	4.9	14	2.1	2	3.5
PDT reaction										
Erythema (*n* = 1868)										
None	8	1.5	1	0.7	13	2.3	6	1.1	1	2
Mild	124	22.7	42	30.2	144	25.5	77	13.6	11	21.6
Moderate	342	62.5	88	63.3	380	67.3	406	71.7	32	62.7
Severe	73	13.3	8	5.8	28	5	77	13.6	7	13.7
Presence of urticaria	221	38.6	50	31.8	220	33.7	384	52.3	34	59.6
Median pain score, IQR (*n* = 2047)	5	2.5, 7	5	3, 7.1	5	2, 7	3	1, 5.7	3.5	1.7, 6

Abbreviations: AK, actinic keratoses; ALA, 5‐aminolaevulinic acid; BCC, basal cell carcinoma; BD, Bowen's disease; MAL, methyl aminolevulinate; ne‐ALA, nanoemulsion ALA; Nod, nodular; PDT, photodynamic therapy; Sup, superficial.

Of the 572 AK‐treated lesions, the most common AK treatment site was head/neck (57.9%). The mean number of treatments ± standard deviation (SD) was 1.7 ± 1.1. The median pain score (IQR) reported for maximal pain experienced during PDT was 5 (2.5, 7) (Table 1). Clearance at 3 months and 12 months after the first cPDT cycle was achieved in 58.1% and 53.4% of AK lesions, respectively (Figure 1).

In this cohort, we treated 157 Bowenoid AK lesions. The most frequently treated lesion/field site was the extremities (56.5%). The mean number ± SD of PDT treatments was 2.6 ± 1.1. The median pain score (IQR) was 5 (3, 7.1). Clearance at 3 months and 12 months after the first PDT cycle was achieved in 55.7% and 71.3% of Bowenoid AK lesions, respectively.

**FIGURE 2:**
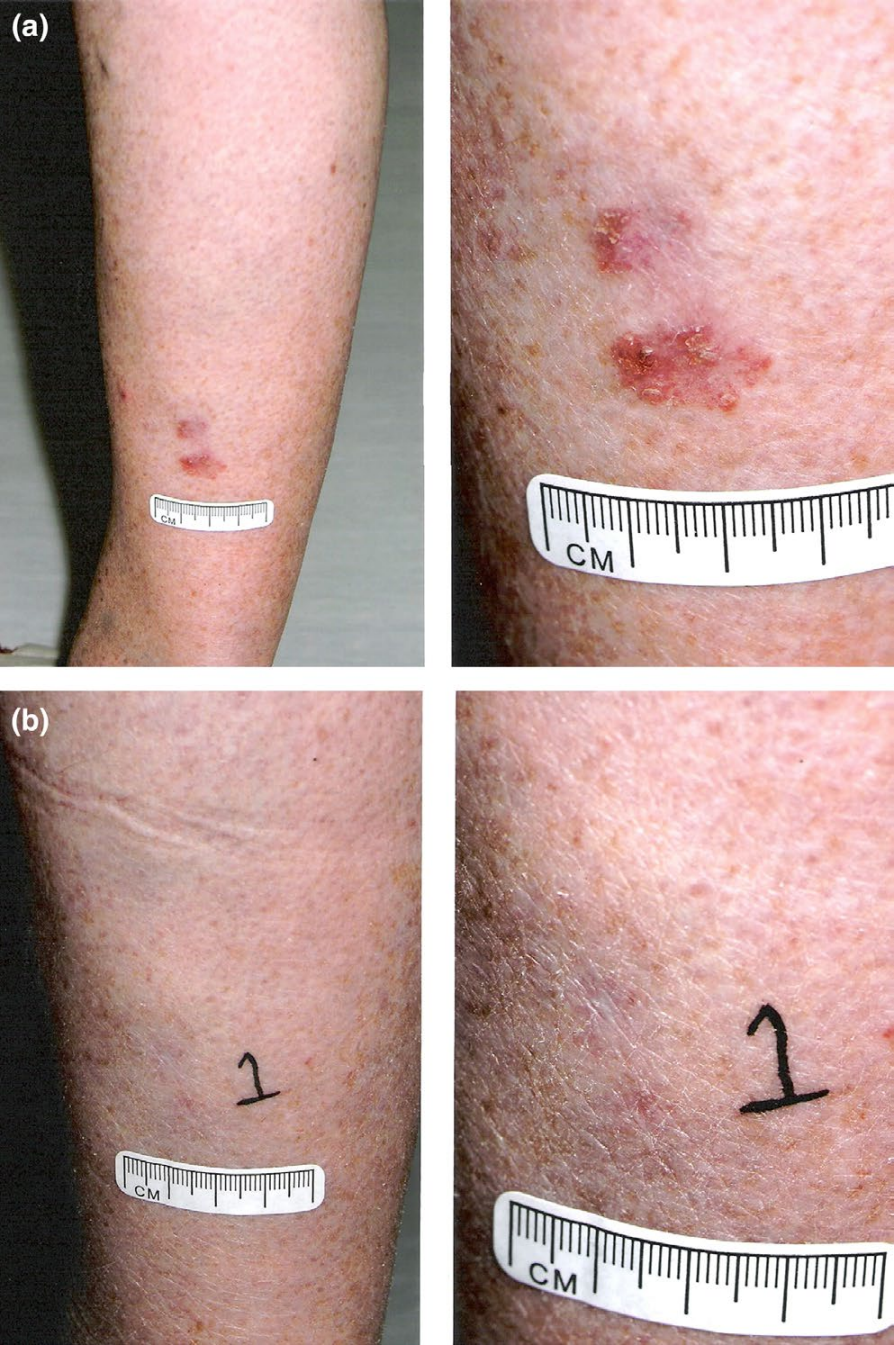
(a) Bowen's disease on the leg before PDT treatment with a close‐up view of the lesion. (b) Complete clearance of Bowen's disease was achieved after four PDT treatments, with a close‐up view of the lesion.

Data derived from 652 BD lesions treated by cPDT were analysed. The majority of BD lesions were located on the extremities (91.8%). The mean number ± SD of PDT treatments was 2.7 ± 1.2, and the median pain score (IQR) reported was 5 (2, 7). Clearance at 3 months and 12 months after the first PDT cycle was achieved in 69% and 86.4% of BD lesions, respectively. Figure 2a,b and show the BD lesion before treatment and its complete clearance after four PDT treatments at the 15‐month follow‐up.

Of our patient cohort, 670 superficial BCC lesions were treated with cPDT. The most common lesion site was the trunk (56.8%). The median histological Breslow tumour thickness (IQR) was 0.3 (0.2, 0.5) mm. The mean number of PDT treatments ±SD was 2.7 ± 1.1. The median pain score was the lowest [3, (IQR 1.5, 7)] compared to other diagnoses (Table 1). Clearance at 3 months and 12 months after the first PDT cycle was achieved in 74.7% and 89% of superficial BCC lesions, respectively.

A total of 57 nodular BCC lesions were included. The most common lesion site was the trunk (41.8%). The median histological tumour thickness (IQR) was 0.9 (0.7, 1.3) mm. The mean number of PDT treatments was the highest (3.2 ± 1.2) compared with other diagnoses treated with cPDT. The median pain score (IQR) reported was 3.5 (1.7, 6) (Table 1). Clearance of nodular BCC lesions at 3 months and 12 months was achieved in 52.1% and 89.7%, respectively.

When determining the response (clearance at 12 months), multivariate analyses revealed that small lesion size was associated with a better outcome for several diagnoses, including AK (odds ratio; OR = 0.81), Bowenoid AK (OR = 0.76) and BD (OR = 0.83) (Table 2). The presence of a thinner histological tumour thickness was a positive prognostic factor for response to cPDT in superficial BCC (OR = 0.29). Surprisingly, the presence of an urticarial reaction immediately after cPDT appeared to be a negative prognostic factor for response to PDT in AK (OR = 0.29). The correlation between pain score and cPDT treatment outcome was inconsistent, with pain score being directly correlated to the outcome for BD (OR = 1.14) but with the reverse being seen for nodular BCC (OR = 0.51).

**TABLE 2:** Multivariate analyses for factors associated with clear outcome at 12 months and moderate to severe pain (pain score ≥ 4).

	Odd ratio	95% CI	*p*
Clear outcome at 12 months			
Lesion size			
Actinic keratoses	0.81	0.71–0.92	0.001
Bowenoid actinic keratoses	0.76	0.67–0.87	< 0.001
Bowen's disease	0.83	0.76–0.92	< 0.001
Histological tumour thickness			
Superficial basal cell carcinoma	0.29	0.10–0.85	0.023
Urticarial reaction (Yes vs. No)			
Actinic keratoses	0.29	0.11–0.74	0.01
Pain score			
Bowen's disease	1.14	1.03–1.25	0.01
Nodular basal cell carcinoma	0.51	0.28–0.95	0.034
Moderate to severe pain			
Sex (female vs. male)			
Bowenoid actinic keratoses	6.14	2.28–16.55	< 0.001
Bowen's disease	3.64	1.98–6.66	< 0.001
Superficial basal cell carcinoma	1.69	1.11–2.56	0.014
Nodular basal cell carcinoma	7.32	1.47–36.40	0.015
Lesion size			
Actinic keratoses	1.16	1.08–1.24	< 0.001
Site (head vs. trunk)			
Actinic keratoses	12.24	5.33–28.14	< 0.001
Superficial basal cell carcinoma	2.42	1.38–4.22	0.002
(Ext vs. trunk)			
Actinic keratoses	5.21	2.21–12.30	< 0.001
Superficial basal cell carcinoma	1.56	1.02–2.40	0.042
Fluorescence intensity (grading 0–3)			
Bowen's disease	1.58	1.23–2.04	< 0.001
Superficial basal cell carcinoma	1.59	1.24–2.02	< 0.001
Fluorescence specificity (yes vs. no)			
Bowenoid actinic keratoses	8.80	1.73–44.87	0.009
Erythema reaction (grading 0–3)			
Actinic keratoses	2.40	1.63–3.53	< 0.001
Bowenoid actinic keratoses	5.39	2.11–13.74	< 0.001
Bowen's disease	2.62	1.72–3.98	< 0.001
Urticarial reaction (yes vs. no)			
Nodular basal cell carcinoma	11.40	2.24–58.02	0.003

Abbreviations: ALA, 5‐aminolaevulinic acid; MAL, methyl aminolaevulinate; ne‐ALA, nanoemulsion ALA.

For moderate to severe pain (pain score ≥ 4) experienced with cPDT, multivariate analysis indicated that there were several associated factors (Table 2). Females were significantly more likely to experience moderate to severe pain during cPDT for several diagnoses, including Bowenoid AK (OR = 6.14), BD (OR = 3.64), superficial BCC (OR = 1.69) and nodular BCC (7.32). If lesions were at a head/neck location, followed by extremities, there was a significantly higher likelihood of experiencing moderate to severe pain in AK (OR = 12.24/5.21) and superficial BCC (OR = 2.42/1.56), when compared to lesions on the trunk. For AK, larger lesion/field size (OR = 1.16) was slightly statistical significance associated with moderate to severe pain. Higher fluorescence intensity was significantly associated with pain in BD (OR = 1.58) and superficial BCC (OR = 1.59). Fluorescence specificity was highly associated with pain in Bowenoid AK (OR = 8.80). More intense PDT‐induced erythema was significantly associated with pain in AK (OR = 2.40), Bowenoid AK (OR = 5.39) and BD (OR = 2.62). The presence of an urticarial reaction after cPDT were associated with greater likelihood of moderate to severe pain for nodular BCC (OR = 11.40).

**Daylight PDT:** This study included 77 patients with field change AK who underwent 158 treatment courses of dPDT (Table 3). The majority of these patients were male (92.4%) and treatment was mainly on head/neck sites (69%). Excellent or good treatment outcomes were reported in 63.3% of cases. The median pain score (IQR) was 1.5 (0.6, 3.2). The majority of patients rated satisfaction with their dPDT outcome as excellent or good (91%) and considered dPDT to be better than previous AK therapies (61.3%). Moreover, 93.6% of patients preferred dPDT to cPDT. No statistical differences were found between the efficacy, adverse effects and pain scores of lesions located on the head/neck or trunk/extremities. Additionally, we observed that a higher visible light exposure dose correlated with higher chances of an excellent outcome. Specifically, patients exposed to a higher light dose (15.4 J/cm^2^) were more likely to have an excellent outcome compared to those who received a lower dose (11.9 J/cm^2^) (*p* = 0.016) (Table 4).

**TABLE 3:** Demographic data, treatment protocol and outcome for actinic keratoses receiving daylight PDT.

	All patients		Head and neck		Trunk & extremities		*p*
	77 patients with 158 treatment courses		75 patients with 109 treatment courses		33 patients with 49 treatment courses		
	*n*	%	*n*	%	*n*	%	
Demographic data							
Sex							
Female	12	7.6	7	6.4	5	10.2	0.517
Male	146	92.4	102	93.6	44	89.8	
Mean age (years), SD	73.6	8.6	73.8	8.7	73.0	8.2	0.509
Treatment protocol							
Photosensitizer							
MAL	148	93.7	99	90.8	49	100	0.032
Ne‐ALA	10	6.3	10	9.2	—	—	
Median PpIX‐weighted light dose (J/cm^2^), IQR (*n* = 168)	13.4	9.4, 19	14	9.6, 19.4	12.2	9.1, 17.8	0.584
Mean number of treatments per year, SD	2.9	0.8	2.9	0.8	2.9	0.7	0.859
Treatment outcome							
Excellent	46	29.1	35	32.1	11	22.4	0.452
Good	54	34.2	37	33.9	17	34.7	
Moderate	43	27.2	26	23.9	17	34.7	
Slight/no response	15	9.5	11	10.1	4	8.2	
Adverse effect							
Median pain score, IQR	1.5	0.6, 3.2	1.5	0.5, 3.3	1.4	0.7, 3.1	0.960
Patient response to PDT							
Patient rated treatment (*n* = 133)							
Excellent	69	51.9	50	54.3	19	46.3	0.694
Good	52	39.1	33	35.9	19	46.3	
Fair	11	8.3	8	8.7	3	7.3	
Poor	1	0.8	1	1.1	—	—	
Compared daylight PDT with any other previous treatment (*n* = 111)							
Better	68	61.3	46	60.5	22	62.9	0.715
Similar	36	32.4	26	34.2	10	28.6	
Worse	7	6.3	4	5.3	3	8.6	
Prefer conventional or daylight PDT (*n* = 110)							
Daylight PDT	103	93.6	70	94.6	33	91.7	0.681
Conventional PDT	7	6.4	4	5.4	3	8.3	

Abbreviations: ALA, 5‐aminolaevulinic acid; MAL, methyl aminolaevulinate; ne‐ALA, nanoemulsion ALA; PDT, photodynamic therapy; PpIX, protoporphyrin IX.

**TABLE 4:** Factors associated with excellent outcomes in daylight PDT.

	Excellent outcome, *N* = 46		Other outcome, *N* = 112		*p*
	*n*	%	*n*	%	
Factors					
Sex					
Female	3	6.5	9	8	1.00
Male	43	93.5	103	92	
Mean age (years), SD	73.8	7.0	73.5	9.1	0.890
Site					
Head and neck	35	76.1	74	66.1	0.216
Trunk and extremities	11	23.9	38	33.9	
Photosensitizer					
MAL	42	91.3	106	94.6	0.478
Ne‐ALA	4	8.7	6	5.4	
Median PpIX‐weighted light dose (J/cm^2^), IQR (*n* = 168)	15.4	11.5, 21.9	11.9	8.9, 17.9	0.016
Mean number of treatments per year, SD	2.9	0.9	2.9	0.8	0.995
Median pain score, IQR	1.2	0.2, 2.9	1.9	0.6, 3.3	0.093

## 4 Discussion

This study revealed the outcomes of topical PDT in a real‐life clinical setting within a PDT clinic in a teaching hospital. As anticipated, efficacy outcomes were not as high as have been shown in controlled trial studies, in which immunocompetent patients are typically highly selected, based on having single, small number, superficial and previously untreated lesions [10, 11, 12, 13]. As a regional PDT service, patients referred to us for PDT usually have multiple lesions/fields, moderate‐to‐severe disease and/or have failed to respond/recurred after other treatments and thus aiming for such high complete clearance rates may not be realistic. Although a previous prospective trial study reported a response rate of 70%–90% for facial and scalp AK [14], our study showed that clearance was only achieved in around 50% of patients receiving cPDT and excellent/good outcomes were reached in around 60% of patients receiving dPDT. In the real‐life setting field change AK is a chronic progressive disease, with the likelihood of complete and maintained clearance being low. Furthermore, these patients typically have subclinical disease in extensive background dysplasia. As the rates of AK transformation to squamous cell carcinoma range from 0% to 0.075% per lesion‐year [15], we consider that patient and clinician expectations of chronic disease management, with recognition of the expected need for repeated and rotated treatments, might be a more pragmatic approach.

In keeping with previous PDT studies, we showed that smaller lesions (for AK, Bowenoid AK and BD) and thinner BCCs are more likely to clear with cPDT [16, 17]. Despite the enhanced lipophilicity of MAL compared to ALA [18], there was no significant difference in cPDT outcomes between the pro‐drugs used. Furthermore, lesion body site did not impact on cPDT outcomes. Similarly, there was no significant correlation between irradiation dose and cPDT outcomes and inconsistent associations between pain scores and cPDT outcomes. Notably, in univariate analyses, an inverse relationship was observed between the number of cPDT treatments and the likelihood of achieving clearance for Bowenoid AK, BD, and superficial BCC, possibly because complex lesions require more treatments for clearance. However, treatment number was not included in further multivariate analyses to prevent confounding.

The outcomes of PDT in immunosuppressed patients were expected to be inferior to those in immunocompetent patients [19], both due to cutaneous immunosuppression and the more hyperkeratotic AK in organ transplant patients [20]. In our study, 4.9%–15.7% of patients, depending on diagnosis, were immunosuppressed (Table 1). Clearance rates at 12 months were not significantly lower in these patients compared to the immunocompetent, for Bowenoid AK (63.6% vs. 71.2%), BD (73.3% vs. 86.8%) and superficial BCC (87.5% vs. 90.1%) (*p* > 0.05). However, as this was a retrospective study, further prospective, controlled trials are required [19, 20].

The main adverse effect was pain. The mechanisms of PDT‐induced pain are not fully understood, but are thought to involve neuropathic and inflammatory pathways [4]. The head/neck location showed significant association with moderate to severe pain, possibly because this area is highly innervated [21]. Higher levels of pain were observed in lesions with stronger fluorescence, likely reflecting enhanced photosensitiser accumulation, which also reflects the association with higher intensity of erythema and an urticarial reaction after PDT. Although female sex was strongly associated with moderate to severe pain in our study, other studies have shown conflicting results with respect to sex, with some demonstrating that males experienced higher levels of PDT‐induced pain, while others found no significant impact of sex on PDT‐induced pain [21, 22].

Our study did not support previous reports of lower pain levels with MAL PDT than with ALA PDT, although different mechanisms may be involved depending on the pro‐drug used. ALA is taken up by beta‐amino acid and γ‐aminobutyric acid transporters (GABA), which may explain the neurogenic pain in ALA PDT, whereas MAL is taken up by mechanisms involving nonpolar amino acids [23]. Furthermore, ALA is associated with higher levels of protoporphyrin IX accumulation, fluorescence intensity and phototoxic reactions than MAL [23]. However, previous studies comparing pain between MAL and ALA PDT are conflicting [24]. Pain in PDT is associated with multiple factors and pro‐drug choice may not be the single determining factor.

For cPDT, the frequency of requesting fan/cold air cooling or local anesthetic drugs as pain‐relieving methods ranged from 10.5% to 19.7% and 2.1% to 10.2%, respectively, across the different diagnoses (Table 1). Patients who requested pain relief typically reported high pain scores after completing the cPDT session, indicating the relative inefficacy of these methods. To avoid bias, the multivariate of pain in cPDT did not include these methods as variable.

In this study we observed good therapeutic outcomes after dPDT for AK, with much lower pain scores than for cPDT. dPDT operates on the principle of continuous, low‐level daylight activation of photosensitizer, in contrast to the rapid activation and depletion seen in conventional red‐LED PDT. The minimal pain experienced with dPDT aligns with prior studies that highlight its nearly painless procedure [4]. Most of our patients rated dPDT satisfaction as good or excellent, and more than 90% preferred dPDT to cPDT.

A previous study has found that the efficacy of dPDT is not affected by the total dose of light exposure, as long as a critical threshold of 8 J/cm^2^ is met. This suggests that efficacy of dPDT is independent of light dose above this threshold [4, 25]. In our study, the median protoporphyrin IX‐weighted light dose was 13.4 J/cm^2^, and 87.3% of participants received a dose of ≥ 8 J/cm^2^. This suggests that daylight exposure levels in Scotland from April to October are usually adequate for successful dPDT. However, we observed that higher light doses above 8 J/cm^2^ were associated with better outcomes in dPDT and further prospective studies are required to clarify this.

One limitation of this study is that it was retrospective, with variables in the PDT protocol during the study period, although this reflects real‐life use. Additionally, long‐term follow‐up and lesion recurrence data were lacking, which should be included in future prospective studies.

In summary, this study provides valuable insights into the effectiveness and practicality of use of PDT for cutaneous dysplasia and NMSC. In a real‐life setting, the success rate of PDT may be lower than expected based on study evidence, especially for AK. However, focusing on chronic disease management could be a more practical approach than aiming for the unrealistic outcomes of complete and maintained AK clearance. dPDT offers the opportunity for an effective but less painful procedure and can be performed in the patient's home environment. The identification of factors associated with PDT treatment outcomes and pain may assist clinicians with respect to optimisation and personalisation of PDT regimens for individual patients.

## Author Contributions

C.C. and S.H.I.: conceptualization/design and methodology of the study; A.L.: data collection; C.C.: data analysis, writing original draft of the manuscript; S.H.I.: supervision and project administration, writing and editing the manuscript. R.D.: editing and reviewing the manuscript.

## Ethics Statement

This study was approved by the NHS Tayside Caldicott Guardian Committee (IGTCAL11264).

## Conflicts of Interest

S.H.I. received support from Galderma to attend conferences and a small grant for a home‐PDT project for the department. The other authors declare no conflicts of interest.

## Supporting information


**Figure S1.** Pre‐treatment fluorescence intensity and specificity assessment of the basal cell carcinoma.

## Data Availability

The data that support the findings of this study are available from the corresponding author upon reasonable request.
