# Supplementary figures and images for: Topical Photodynamic Therapy in a Medical Centre: The Scottish Dermatology Experience
Source: Photodermatol Photoimmunol Photomed. 2025 Feb 3;41(2):e70010. doi: 10.1111/phpp.70010 (PMC11790065; doi:10.1111/phpp.70010)

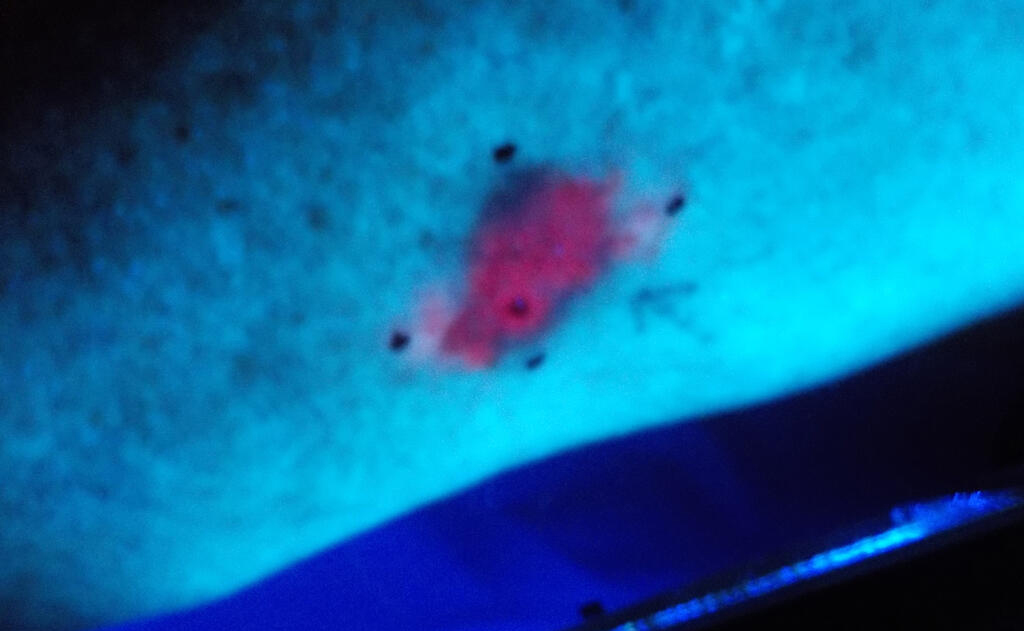

Supplement: Supplementary file 1 — Figure S1. Pre‐treatment fluorescence intensity and specificity assessment of the basal cell carcinoma. [file PHPP-41-e70010-s001.jpg]
